# ‘Harlequin cells’ in lymphocyte‐variant hypereosinophilia

**DOI:** 10.1002/jha2.576

**Published:** 2022-10-10

**Authors:** Amirali Vahedi, Tahereh Madani, Behrooz Gharib, Behzad Poopak

**Affiliations:** ^1^ Payvand Clinical, Specialty, Pathology Medical Genetics and Molecular Laboratory Tehran Iran; ^2^ Oil Hospital Tehran Iran; ^3^ Islamic Azad University Tehran Medical Sciences Tehran Iran

**Keywords:** flow cytometry, lymphocyte‐variant hypereosinophilia, T‐cells

1

A 36‐year‐old woman presented to her physician with a 2‐month history of fatigue and swollen hands and feet. Her complete blood count (performed at another center) showed normal hemoglobin (125 g/l) and platelet count (222 × 10^9^/l) with increased white blood cell (WBC) count (15.9 × 10^9^/l). Upon WBC differential count, she was found to have a significant increase in eosinophils (53%). A bone marrow aspiration was referred to our laboratory for the evaluation of eosinophilia. Cytomorphology examination showed 2% blasts and 43% dysplastic eosinophils and their precursors including myelocyte and metamyelocyte stages. These atypical eosinophils had large violet granules similar to granules seen in basophils (Figure 1A, Thiazine‐Eosin; panel A; ×100 objective). *PDGFRA* and *PDGFRB* rearrangements were evaluated for the patient by fluorescence in situ hybridization and were negative. Polymerase chain reaction was performed for *t(9;22)*, *inv(16)* and *t(16;16)* and was negative for all. Patient also had normal karyotype 46,XX. Flow cytometric immunophenotyping was performed for the exclusion of lymphocyte‐variant hypereosinophilia (Figure 1B). Normal myeloblasts and B‐cell precursors (hematogones) were present. Eosinophils (about 43% of total nucleated cells) showed no aberrant expression of myeloid antigens. Upon investigating lymphocytes, an abnormal population was observed (panel B). It comprised 0.06%, 0.80% and 1.21% of total nucleated cells, lymphocytes and T‐cells, respectively. The immunophenotype of this population was CD2+, cytoplasmic CD3+, CD3‐, CD4+, CD5 bright, CD7‐/dim, CD8‐, CD45 bright, CD56‐ and TCRαβ‐. Based on immunophenotyping and cytogenetics, a diagnosis of lymphocyte‐variant hypereosinophilia was made. Patient was treated with corticosteroids, and her symptoms and eosinophilia were resolved. A wait‐and‐see approach with extended follow‐up aim at the abnormal T‐cell population was adopted.

**FIGURE 1:**
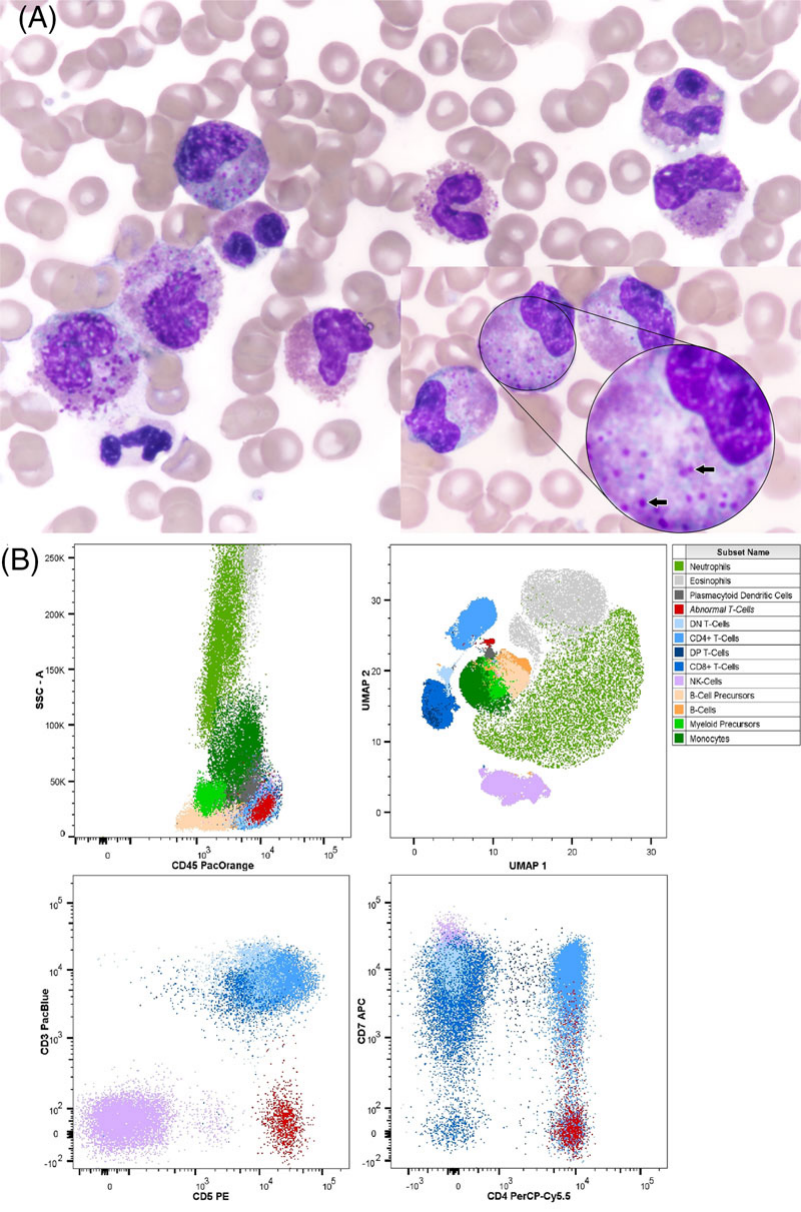
(A) Bone marrow aspiration (Thiazine‐Eosin stain ×100 objective). (B) Flow cytometry immunophenotyping showing the presence of an abnormal T‐cell population in red

Lymphocyte‐variant hypereosinophilia (L‐HES) is a combination of both a reactive, and a clonal condition in which eosinophilia is secondary to the expansion of an abnormal, cytokine‐producing T‐cell population [1]. The eosinophilia is not associated with myeloid malignancies and is a result of the production of eosinophilopoietic cytokines, such as IL‐5, by clonal T‐cells [1]. Flow cytometric immunophenotyping is the cornerstone of the diagnosis of this entity.

Dysplastic eosinophils containing both eosinophilic and basophilic granules in their cytoplasms are called ‘Harlequin cells’ or hybrid eosinophil/basophils [2]. These cells are generally thought to be associated with myeloid malignancies such as acute myeloid leukaemia (AML; specifically, AML with *inv(16)* or *t(16;16)*) and chronic myeloid leukaemia [2]. However, recent studies suggest that these immature eosinophils are in fact not specific to any type of haematopoietic malignancies and can even be seen in normal or reactive bone marrow [3]. To our knowledge, no case report has ever been published demonstrating the presence of ‘harlequin cells’ in the setting of L‐HES. This is the first report of this phenomenon emphasizing that the dysplastic presentation of ‘harlequin cells’ should not prompt the exclusion of L‐HES, and performing flow cytometry for the screening of abnormal T‐cells would be still recommended.

## AUTHOR CONTRIBUTIONS

AV and TM analysed the data, created the figures and wrote the paper. BP and BG designed the research study and wrote the paper. BP supervised the project.

## CONFLICT OF INTEREST

The authors declared no conflict of interest.

## FUNDING INFORMATION

This study received no specific grant from any funding agency in the public, commercial or non‐profit sectors.

2

## ETHICS STATEMENT

This is an original case report, has not been previously published and has not been submitted for publication anywhere else, and will not be published elsewhere without journal consent. Written informed consent was obtained from the patient reported in this study.

## Data Availability

Data sharing is not applicable to this article as no new data were created or analysed in this study.

## References

[1] Shomali W , Gotlib J . World Health Organization‐defined eosinophilic disorders: 2019 update on diagnosis, risk stratification, and management. Am J Hematol. 2019;94(10):1149–67. 31423623 10.1002/ajh.25617

[2] Hirano T , Eto K . Harlequin cells. Blood. 2018;132(7):766. 30115636 10.1182/blood-2018-05-852095

[3] Jain G , Kumar C , Chopra A . Harlequin cell: ubiquitous or pathognomic? Int J Lab Hematol. 2020;42(2):e42–4. 31424608 10.1111/ijlh.13092

